# Supplementary material for: Intensive Care Management of Severe Hyponatraemia—An Observational Study
Source: Medicina (Kaunas). 2024 Aug 29;60(9):1412. doi: 10.3390/medicina60091412 (PMC11434366; doi:10.3390/medicina60091412)
Supplement: Supplementary file 1 [file medicina-60-01412-s001.zip › medicina-3170974-supplementary.pdf]

Supplementary Material

Supplementary material Table S1: Missing data from baseline characteristics.

Characteristic	Total Missing (n=181)	Normal Corrected Missing (n=119)	Overcorrected Missing (n=62)
Alcohol Excess	1	0	1
Baseline Creatinine (μmol/L)	5	4	1
Neurological Insult	1	1	0
Serum Osmolarity (mOsm/kg)	34	21	13
Urinary Sodium (mEq/L)	68	44	24
TSH (miU/L)	58	43	15
T4 (pmol/L)	74	48	26
Cortisol (nmol/L)	66	44	22

Supplementary material Table S2: number of pateints meeting each criteria of overcorrection within 48 hours definition

Overcorrection definition	Number of Patients
Within 24 hours (>10mmol/L)	33 (17%)
From 24-48 hours (>8mmol/L)	32 (17%)
From 0-48 hours (>18mmol/L	22 (12%)
Combined definition	62 (34%)
Multiple criteria met	42 (23%)

Supplementary material Table S3: Univariate analysis for risk of overcorrection within 48 hours.

Characteristic	Odds ratio	95% Confidence Interval	p-value
Admission Sodium Level Value	0.91	0.86, 0.96	0.001
Age	0.99	0.97, 1.01	0.2
Male	1.01	0.55, 1.88	>0.9
Chronic Kidney Disease	1.40	0.40, 4.59	0.6
Symptomatic Hyponatremia	1.51	0.77, 3.05	0.2
Diuretics	0.61	0.31, 1.20	0.2
Vaptans	0.96	0.13, 5.06	>0.9
Fluid restriction	0.54	0.29, 1.01	0.056
Hypertonic NaCl Infusion	1.17	0.63, 2.17	0.6
